# Effects of Targeted Hypercapnia on Mortality and Length of Stay of Post-cardiac Arrest Patients: A Systematic Review and Meta-Analysis

**DOI:** 10.7759/cureus.60617

**Published:** 2024-05-19

**Authors:** Nanush Damarlapally, Tanya Sinha, Anurag Rawat, Thin M Soe, Ghazala Munawar, Sandipkumar S Chaudhari, Calvin R Wei, Neelum Ali

**Affiliations:** 1 Health Sciences, Coleman College of Health Sciences, Houston, USA; 2 Medicine, Tribhuvan University, Kathmandu, NPL; 3 Interventional Cardiology, Himalayan Institute of Medical Sciences, Dehradun, IND; 4 Medicine, University of Medicine 1, Yangon, MMR; 5 Internal Medicine, Northwest General Hospital and Research Center, Peshawar, PAK; 6 Cardiothoracic Surgery, University of Alabama at Birmingham, Birmingham, USA; 7 Family Medicine, University of North Dakota School of Medicine and Health Sciences, Fargo, USA; 8 Research and Development, Shing Huei Group, Taipei, TWN; 9 Internal Medicine, University of Health Sciences, Lahore, PAK

**Keywords:** systematic review and meta-analysis, normocapnia, length of stay, mortality, hypercapnia

## Abstract

Therapeutic hypercapnia has been proposed as a potential strategy to enhance cerebral perfusion and improve outcomes in patients after cardiac arrest. However, the effects of targeted hypercapnia remain unclear. We conducted a systematic review and meta-analysis to evaluate the impact of hypercapnia compared to normocapnia on mortality and length of stay in post-cardiac arrest patients. We searched major databases for randomized controlled trials and observational studies comparing outcomes between hypercapnia and normocapnia in adult post-cardiac arrest patients. Data on in-hospital mortality and the ICU and hospital length of stay were extracted and pooled using random-effects meta-analysis. Five studies (two randomized controlled trials (RCTs) and three observational studies) with a total of 1,837 patients were included. Pooled analysis showed hypercapnia was associated with significantly higher in-hospital mortality compared to normocapnia (56.2% vs. 50.5%, OR 1.24, 95% CI 1.12-1.37, p<0.001). There was no significant heterogeneity (I2 = 25%, p = 0.26). No statistically significant differences were found for ICU length of stay (mean difference 0.72 days, 95% CI -0.51 to 1.95) or hospital length of stay (mean difference 1.13 days, 95% CI -0.67 to 2.93) between the groups. Sensitivity analysis restricted to mild hypercapnia studies did not alter the mortality findings. This meta-analysis did not find a mortality benefit with targeted hypercapnia compared to normocapnia in post-cardiac arrest patients. The results align with current guidelines recommending a normal partial pressure of arterial carbon dioxide (PaCO2) target range and do not support routinely targeting higher carbon dioxide levels in this setting.

## Introduction and background

Cardiac arrest stands as a significant health issue, with an annual occurrence of approximately 50 to 110 per 100,000 individuals globally [1]. Despite effective cardiopulmonary resuscitation (CPR) initially, a notable portion of survivors of cardiac arrest either pass away before leaving the hospital or suffer from severe neurological damage [2]. Following the restoration of spontaneous circulation (ROSC), the initial hours and days constitute what is termed the post-cardiac arrest syndrome, often representing the epitome of critical illness and carrying a high risk of morbidity and mortality [3]. The management of post-cardiac arrest necessitates meticulous monitoring and intervention [4]. 

Arterial carbon dioxide tension (PaCO2) plays a pivotal role in determining cerebral blood flow and possesses anticonvulsive, anti-inflammatory, and antioxidant attributes, rendering therapeutic hypercapnia a promising avenue for enhancing cerebral perfusion post-cardiac arrest [5-6]. International guidelines advocate for targeting normocapnia in comatose adults resuscitated after out-of-hospital cardiac arrest [7-8]. Nevertheless, normocapnia may prove inadequate for restoring and sustaining adequate cerebral perfusion. Two observational studies indicated that, after adjusting for illness severity, exposure to hypercapnia correlated significantly with higher odds of being discharged home and achieving improved neurological outcomes at 12 months, in comparison to hypocapnia or normocapnia [9-10]. 

Nevertheless, there remains a dearth of comprehensive evidence concerning the effectiveness of hypercapnia, specifically in adult post-cardiac arrest. Therefore, it is imperative to undertake a meta-analysis to amalgamate existing data on this subject. Through systematic review and analysis of pertinent studies, this meta-analysis endeavors to furnish a thorough comprehension of the repercussions of mild hypercapnia on outcomes in cardiac arrest patients. The outcomes of this investigation have the potential to influence clinical practice guidelines and enhance patient management during the critical post-cardiac arrest phase, ultimately resulting in improved outcomes and decreased morbidity and mortality rates.

## Review

Methodology 

Search Strategy 

A thorough exploration of the literature was conducted across various electronic databases, including PubMed, Embase, the Cochrane Central Register of Controlled Trials (CENTRAL), and Web of Science. The search strategy entailed a combination of pertinent keywords and medical subject headings (MeSH) terms pertaining to "cardiac arrest," "hypercapnia," "carbon dioxide," and "therapeutic." The search was confined to studies involving human participants and published in the English language. Furthermore, the reference lists of the included studies and relevant review articles were manually scrutinized to identify any additional eligible studies. The search process was carried out independently by two authors, and any discrepancies between them were resolved through discussion.

Study Selection 

Two independent reviewers conducted the study selection process. The titles and abstracts of the identified studies were screened according to predefined inclusion and exclusion criteria. Any discrepancies between the reviewers were resolved through discussion or, if needed, consultation with a third reviewer. The full texts of potentially eligible studies were then retrieved and subjected to further assessment for inclusion. 

We included randomized controlled trials (RCTs) or observational studies (cohort, case-control, or cross-sectional studies) involving adult patients (≥18 years) who experienced cardiac arrest and received post-cardiac arrest care. We included all studies comparing the outcomes of patients exposed to hypercapnia (PaCO2 levels >45 to 60 mmHg) with those exposed to normocapnia. Studies reporting at least one of the following outcomes: all-cause mortality, hospital length of stay (LOS), and ICU LOS were included in this meta-analysis. We excluded studies involving pediatric or neonatal populations. We also excluded studies assessing hypercapnia in non-cardiac arrest settings. Additionally, case reports, case series, reviews, editorials, or conference abstracts were also excluded. 

Data Extraction 

Data from the included studies were extracted independently by two reviewers using a standardized data extraction form. Relevant information, such as study characteristics (design, sample size, setting), participant characteristics (age, gender), and outcome measures, was extracted. Any discrepancies in data extraction were resolved through discussion or consultation with a third reviewer. 

Statistical Analysis 

The meta-analysis was conducted utilizing RevMan version 5.4.1 (The Cochrane Collaboration, The Nordic Cochrane Centre, Copenhagen, Denmark). For dichotomous outcomes such as mortality, pooled odds ratios (ORs) with corresponding 95% confidence intervals (CIs) were computed. For continuous variables, the mean difference was computed with a 95% CI. A significance level of p<0.05 was deemed statistically significant. Heterogeneity among studies was evaluated using Cochran's Q test and the I² statistic. In cases of substantial heterogeneity (I² > 50%), a random-effects model was applied for the meta-analysis; otherwise, a fixed-effects model was utilized. We performed sensitivity analysis by including only those studies that used targeted mild hypercapnia (PaCO2: 45 to 55 mmHg) to assess the effect of this intervention on all-cause mortality.

Results

**Figure 1:**
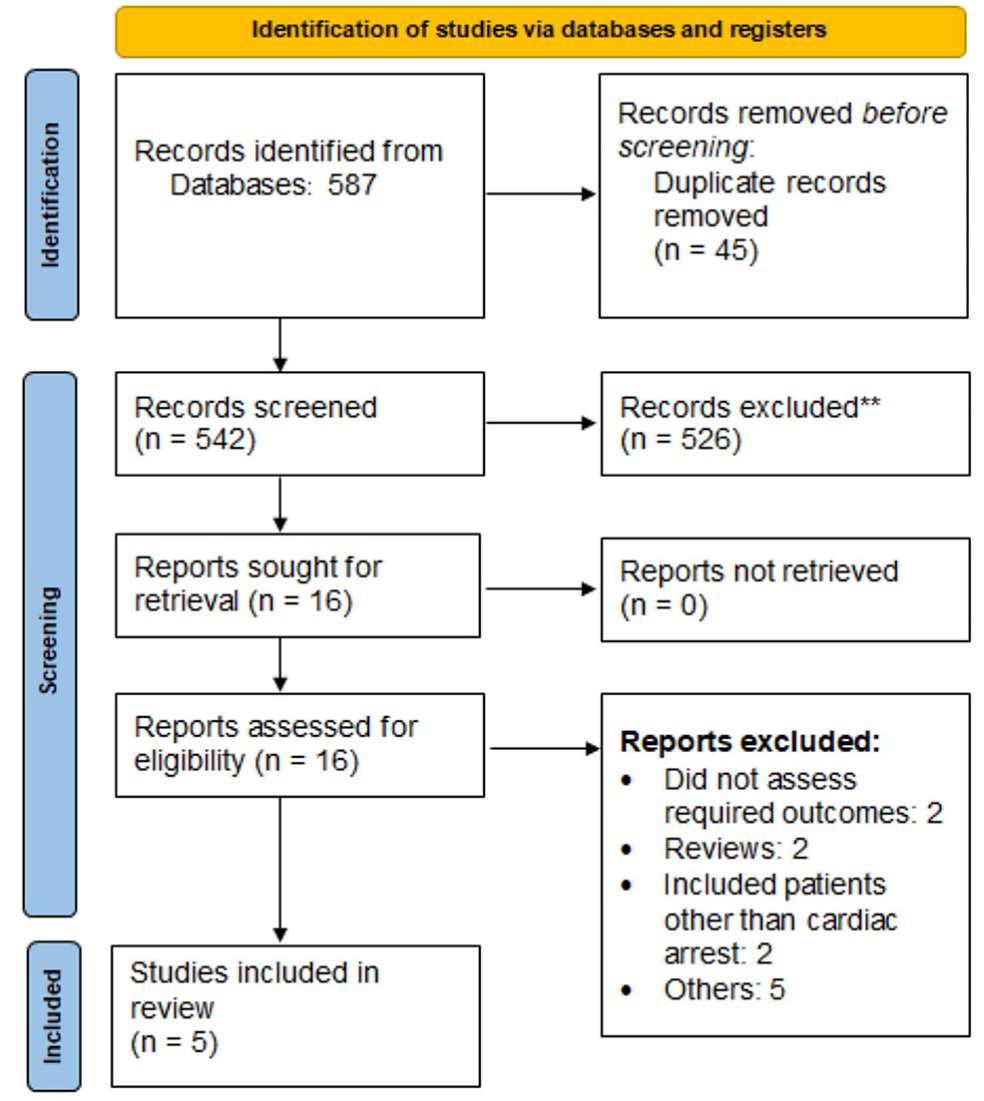
PRISMA flowchart illustrating study selection PRISMA: Preferred Reporting Items for Systematic Reviews and Meta-Analyses

After searching databases, we found 587 records. We removed 45 duplicate studies. Then we screened the remaining 542 articles. Eventually, we selected five studies that met our criteria for inclusion in this analysis. A flowchart of the study selection process is presented in Figure 1. Table 1 displays the characteristics of the included studies. Out of five studies, three were observational and two were RCTs.

**Table 1:** Characteristics of included studies RCT: Randomzied controlled trial

Author ID	Year	Study design	Regions	Groups	No. of participants	Define hypercapnia	Age (years)	Males (n)
Eastwood et al. [11]	2016	RCT	New Zealand	Hypercapnia	42	50 to 55 mmHg	61	33
				Normocapnia	41		61	33
Eastwood et al. [12]	2023	RCT	Multicenter	Hypercapnia	829	45 to 55 mmHg	61.2	635
				Normocapnia	839		61.2	635
Helmerhost et al. [13]	2015	Observational	Netherland	Hypercapnia	1834	>45 mmHg	65	1323
				Normocapnia	2288		66	737
Schneider et al. [9]	2013	Observational	New Zealand	Hypercapnia	6827	>45 mmHg	62.3	4645
				Normocapnia	6705		63.8	4333
Zhou et al. [14]	2020	Observational	China	Hypercapnia	472	45 to 55 mmHg	66	278
				Normocapnia	1088		64	652

Comparison of In-Hospital Mortality Between Hypercapnia and Normocapnia

**Figure 2:**
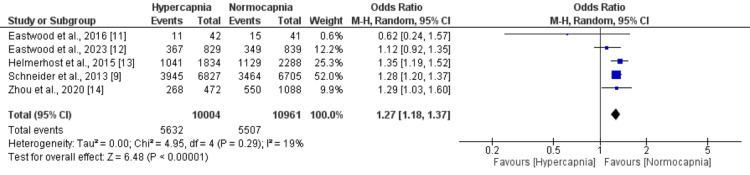
Comparing the risk of mortality between the two groups (hypercapnia vs. normocapnia)

Four studies examined the relationship between hypercapnia and normocapnia in patients who experienced cardiac arrest. The studies compared the risk of in-hospital mortality between these two groups. The combined analysis of the four studies, illustrated in Figure 2, revealed that patients in the hypercapnia group had a significantly higher risk of dying in the hospital compared to those in the normocapnia group. Specifically, 56.3% of patients in the hypercapnia group died in the hospital, while the mortality rate was 50.2% in the normocapnia group. The OR of 1.27 with a 95% CI of 1.18 to 1.37 indicates that the risk of in-hospital mortality was significantly higher for patients with hypercapnia compared to those with normocapnia. The analysis did not find significant heterogeneity among the results of the individual studies, as indicated by an I-square value of 25% and a p-value of 0.26. We performed sensitivity analysis by including those studies with mild hypercapnia, and no significant difference was found in terms of the risk of all-cause mortality (OR: 1.16, 95% CI: 0.96 to 1.40). 

Comparison of ICU and Hospital LOS Between the Hypercapnia and Normocapnia Groups

Table 2 presents the ICU and hospital LOS between the two groups. The mean length of ICU and hospital stays was not significantly different between the two groups (p>0.05). 

**Table 2:** Comparison of ICU and hospital LOS LOS: Length of stay, MD: Mean difference

Outcome	MD (95% CI)	I-square
ICU LOS	-0.05 (-0.38 to 0.28)	0%
Hospital LOS	1.22 (-3.56 to 6.00)	38%

Discussion

The pooled analysis of five studies, including two RCTs and three observational studies, reported that the mortality risk was higher in patients in the hypercapnia group than in patients in the normocapnia group. However, the LOS in the ICU and the LOS in the hospital were not significantly different between the two groups. Thus, we can conclude that the targeted mild hypercapnia did not improve the risk of death in patients with cardiac arrest. The RCT performed by Eastwood et al. [12] also reported that mild hypercapnia did not enhance neurologic outcomes. As far as our knowledge is concerned, this is the first comprehensive meta-analysis comparing the risk of death between targeted hypercapnia and normocapnia in patients with cardiac arrest. 

The impacts of hypercapnia on post-cardiac arrest individuals have been elucidated in numerous studies [10-11,15-16]. Certain studies have demonstrated a relationship between unfavorable clinical outcomes and hypercapnia [13,15], while others have not shown a statistically significant correlation between hypercapnia and neurological outcomes [10-11]. A multicenter RCT focusing on feasibility revealed that targeted therapeutic mild hypercapnia (TTMH) mitigated the release of neuron-specific enolase (NSE) [12]. Nevertheless, there was no discernible association between cerebral oxygenation assessed with near-infrared spectroscopy (NIRS) and NSE concentrations or outcomes in individuals resuscitated from out-of-hospital cardiac arrest [17]. Our meta-analysis revealed that, in comparison to normocapnia, hypercapnia did not yield a higher likelihood of hospital survival. 

Our findings align with the consensus guidelines of 2015 advocating for a normal target range of PaCO2 (35 to 45 mmHg) [18-19]. The study conducted by Zhou et al. highlighted that the proportion of time spent within the normocapnia range was correlated with decreased hospital mortality [14]. Furthermore, the study suggested that the influence of PaCO2 on hospital mortality in post-cardiac arrest patients may be modulated by pH levels [14]. The elusive objective of targeting carbon dioxide levels post-cardiac arrest may need to consider pH levels as well [20]. Given that mortality rates increased with rising PaCO2 levels in post-cardiac arrest patients experiencing hypercapnic acidosis, they did not increase with compensated hypercapnia (normal pH). 

Most of the studies included in this meta-analysis did not differentiate between mild and severe hypercapnia, which may have influenced the overall results. Severe hypercapnia (PaCO2 > 60 mmHg) is generally associated with adverse outcomes, while mild hypercapnia (PaCO2 between 45 and 55 mmHg) has been suggested to have potential benefits in post-cardiac arrest patients [21]. By not separating these two categories of hypercapnia, the potential beneficial effects of mild hypercapnia may have been obscured by the detrimental effects of severe hypercapnia. To address this limitation, we performed a sensitivity analysis by including only those studies that specifically assessed mild hypercapnia (PaCO2 between 45 and 55 mmHg). This sensitivity analysis aimed to provide a more accurate estimate of the effects of mild hypercapnia on mortality in post-cardiac arrest patients. Interestingly, the sensitivity analysis did not find any significant difference between the mortality rates in the normocapnia and mild hypercapnia groups. This finding suggests that, when considering only mild hypercapnia, there is no clear evidence of an increased risk of mortality compared to normocapnia in post-cardiac arrest patients. 

Our meta-analysis and the current guidelines do not support targeted mild hypercapnia as a strategy to improve outcomes in this patient population. Further research is needed to explore the potential role of pH, compensatory mechanisms, and other factors in determining the effects of hypercapnia on outcomes in post-cardiac arrest patients. 

Study Limitations 

The first limitation is that only two RCTs were included. Randomized controlled trials are considered the gold standard for medical research because they help reduce bias. Observational studies, which were not included here, are more prone to bias since they don't randomly assign patients to treatment groups. The authors acknowledge this and emphasize the need for more RCTs to definitively assess the effects of targeted hypercapnia in patients with cardiac arrest. The second limitation is that most of the studies included in the meta-analysis didn't differentiate between mild and severe hypercapnia. This difference may have an effect on the outcome; however, the authors performed an analysis to assess this specifically and did not find a significant impact of mild hypercapnia on mortality. The third limitation is that the researchers couldn't perform a subgroup analysis on factors such as age, gender, and comorbidities due to a lack of data on individual patients. Subgroup analysis is a way to see if the treatment effect is the same across different groups of people. 

## Conclusions

This systematic review and meta-analysis found that targeted mild hypercapnia did not improve survival or reduce hospital LOS in post-cardiac arrest patients compared to normocapnia. These findings align with current guidelines recommending a normal PaCO2 target range of 35 to 45 mmHg in this patient population. However, the role of factors such as compensatory pH changes requires further study. Overall, the results do not provide evidence for adopting targeted therapeutic mild hypercapnia as a strategy to improve outcomes in the post-cardiac arrest setting.
